# Effectiveness of a computer-facilitated intervention on improving provider delivery of tobacco treatment in a thoracic surgery and oncology outpatient setting: A pilot study

**DOI:** 10.18332/tid/186272

**Published:** 2024-04-22

**Authors:** Manan M. Nayak, Emanuele Mazzola, Michael T. Jaklitsch, Jeremy E. Drehmer, Emara Nabi-Burza, Raphael Bueno, Jonathan P. Winickoff, Mary E. Cooley

**Affiliations:** 1 Phyllis F. Cantor Center for Research in Nursing and Patient Care Services, Dana-Farber Cancer Institute, Boston, United States; 2 Department of Psychosocial Oncology and Palliative Care, Dana-Farber Cancer Institute, Boston, United States; 3 Department of Data Science, Dana-Farber Cancer Institute, Boston, United States; 4 Division of Thoracic Surgery, Brigham and Women’s Hospital, Boston, United States; 5 Division of General Academic Pediatrics, Massachusetts General Hospital for Children, Boston, United States; 6 Tobacco Research and Treatment Center, Massachusetts General Hospital, Boston, United States

**Keywords:** tobacco treatment, smoking cessation intervention, thoracic surgery, lung cancer, digital health

## Abstract

**INTRODUCTION:**

Effective tobacco treatments are available but are often not delivered to individuals with an actual or potential diagnosis of thoracic malignancy. The specific aims of this study were to identify the prevalence of tobacco use and examine the effectiveness of the Clinical and community Effort Against Smoking and secondhand smoke Exposure (CEASE), a system-level computer-facilitated intervention, to improve provider delivery of tobacco treatment in a thoracic surgery and oncology outpatient setting.

**METHODS:**

A pre-post-test design was used to assess the effectiveness of CEASE. A 3-step approach was used to integrate tobacco treatment into routine care: ask about tobacco use, assist with cessation, and refer to a quitline. An end-of-visit survey was conducted to collect prevalence of tobacco use and delivery of tobacco treatment. Descriptive statistics and Fisher’s exact test were used for analysis.

**RESULTS:**

A total of 218 individuals were enrolled; 105 participants were in usual care (UC) and 113 were in the CEASE group. Of those who enrolled, 27.6% were never smokers in UC and 27.7% in CEASE, 60% were former smokers in UC and 50% in CEASE, and 12.4% were current smokers in UC and 21.4% in CEASE. Significant differences were noted in delivery of tobacco treatment with 15.4% having received tobacco treatment in UC compared to 62.5% in CEASE (p<0.004).

**CONCLUSIONS:**

A computer-facilitated intervention increased provider delivery of tobacco treatment in a thoracic surgery and oncology outpatient setting. This intervention provided a low-resource approach that has the potential to be scaled and implemented more broadly.

## INTRODUCTION

Smoking cessation is essential among individuals at high-risk of thoracic malignancy to promote lung health, minimize post-operative complications, and improve outcomes during cancer treatment^1^. Combined use of pharmacotherapy and behavioral counseling is one of the most effective treatments for tobacco dependence and can double cessation rates^2^. A common barrier to effective implementation within healthcare settings is lack of a system-level approach that identifies individuals who are current smokers and initiates treatment. A system-level intervention that has increased delivery of tobacco treatment by 12-fold is the Clinical and community Effort Against Smoking and secondhand smoke Exposure (CEASE)^3^. This intervention uses a three-step approach: 1) Ask about tobacco use, 2) Assist individuals through prescribing of cessation medications, and 3) Refer for behavioral counseling.

The objective of this pilot study was to adapt, implement, and test the effectiveness of CEASE, a computer-facilitated tobacco treatment intervention. The specific aims were to: 1) Identify the prevalence of tobacco use among patients in a thoracic surgery and oncology outpatient setting, and 2) Determine the effectiveness of a computer-facilitated intervention in improving provider delivery of tobacco treatment in a thoracic surgery and oncology outpatient setting. Our hypothesis is that the delivery of tobacco treatment (prescription of cessation medications and referral to state quitlines) will be higher after implementation of CEASE, a computer-facilitated intervention.

## METHODS

### Design

A pre-post-test design was used to assess the effectiveness of implementing the CEASE intervention. The protocol was approved by the Dana-Farber Harvard Cancer Center Institutional Review Board (Protocol 16-208).

### Data collection

English-speaking adult patients were consecutively approached prior to their clinical visit and informed consent was obtained to participate in the study at an academic urban thoracic surgery and oncology outpatient setting in the Northeast region of the United States. An end-of-visit survey was administered at pre- and post-implementation, and took between 1 to 5 minutes to complete. Patients reported current tobacco use and receipt of tobacco treatment services, which allowed for comparison of delivery of tobacco treatment before and after implementation of the CEASE intervention.

### Usual care

Prior to CEASE- implementation, we conducted a 4-month observational period to assess the standard tobacco-related assessment and care provided to patients during their appointments in the thoracic surgery and oncologic clinic. This allowed us to establish a baseline understanding of the usual care practices regarding tobacco use evaluation and delivery of tobacco treatment. This pre-implementation phase allowed us to identify strategies for CEASE implementation ensuring easy integration of CEASE intervention within the clinical workflow.

### Intervention

*Implementation of tobacco treatment into the clinical setting*

The Expert Recommendations for Implementing Change taxonomy was used to select strategies to integrate the intervention into the clinical setting^4^. The implementation strategies used during the preparation phase included assessing readiness for the intervention by identifying barriers and facilitators, whereas the strategies that were used to facilitate implementation included adapting and tailoring the intervention for the clinical context, developing stakeholder relationships (e.g. clinicians, front desk, and medical assistants), restructuring the tobacco treatment systems and educating staff. As an initial step, clinical and administrative staff were interviewed to identify the barriers and facilitators for delivery of tobacco treatment. Subsequently, the research team observed and collected data on the workflow of the delivery of treatment services by conducting end-of-visit surveys with patients after their clinical encounters. These data were used to adapt the CEASE intervention and plan for integrating the intervention into routine care.

Next, a clinical champion was identified who collaborated with the research team to facilitate prescription of cessation medications and integrate tobacco treatment into the workflow. Other strategies used to prepare for implementation were to set up a system to collect tobacco use data and initiate treatment^5^. The system included: 1) delivering a survey on an iPad, 2) linking iPad responses to a patient chart, 3) alerting clinicians to patients who were smoking and their preference for receiving tobacco treatment, and 4) facilitating cessation medication and quitline referral by creation of templates in the electronic health records.

In order to prepare for implementation of CEASE, staff training was initiated. The following paragraph explains who was responsible for each step of the CEASE intervention. The first step of CEASE is ‘Ask’. We provided training to clinical staff to facilitate collecting information about tobacco use. Front desk staff training included administering a tobacco survey through an iPad to patients upon check-in to the clinic, reviewing the message displayed on the iPad when patients returned it to them, and flagging patient charts with their tobacco treatment preferences^5^. The second step of CEASE is to ‘Assist’. Medical assistants were trained to review the flagged patient charts, collect additional tobacco history when necessary, and offer pamphlets with information about the state quitline. Clinicians attended educational sessions and received written resources about evidence-based approaches for delivery of tobacco treatment, which included providing brief advice to quit smoking and prescribing cessation medications. The third step of CEASE is ‘Refer’. Clinicians initiated a referral to the state quitline to provide follow-up and initiate behavioral counseling after the clinic visit.

*Computer-facilitated intervention*

The administration of an iPad at the time of check-in became part of routine care. A survey was embedded on an iPad which collected tobacco use data and identified patient preferences for tobacco treatment services^5^. Patients completed this survey within one to five minutes and returned the iPad to the front desk staff. The iPad displayed an additional alert for the front desk to flag patient charts regarding their preference for tobacco treatment, and once this step was completed a new survey was automatically generated. Medical assistants flagged patient charts based on their survey responses for tobacco treatment to alert providers regarding patient preference for tobacco treatment before the visit so that the delivery of tobacco treatment would be integrated as part of routine care.

### Measures

A research team member (not part of the clinic workflow) conducted the end-of-visit survey with patients to assess the pre- and post-implementation of the tobacco treatment intervention. The purpose of the end-of-visit surveys was to obtain patient self-report on whether tobacco use was assessed. Additional information was gathered from individuals who were current smokers, which included whether they received advice to quit smoking, received cessation medication (i.e. nicotine replacement therapy), and a quitline referral.

Demographic variables were obtained through self-report and included age at the time of survey completion, and how they identify their gender, race, and ethnicity, current marital status, education level, current work status, annual household income, and financial situation.

Tobacco use variables were obtained through self-administration of a modified version of the Cancer Patient Tobacco Use Questionnaire (C-TUQ) at the end-of-visit survey^6^. Participants who reported not having smoked at least 100 cigarettes in their entire life (5 packs = 100 cigarettes), were classified as never smokers. Individuals reporting having smoked cigarettes, even a single puff, within the last 30 days, were identified as current smokers. Participants who confirmed having smoked in their lifetime, but had not smoked within the past 30 days, were classified as former smokers. This approach allowed us to clearly delineate the smoking status of participants and analyze the prevalence of tobacco use within our study population.

Tobacco treatment services were assessed through self-report to identify whether tobacco services were offered during the clinical encounter. An end-of-visit survey was used to gather information from patients about whether they were offered tobacco treatment, nicotine patches, nicotine gum, wellbutrin (bupropion) and/or a referral to the quitline.

### Data analysis

Data were summarized using descriptive statistics for demographics, tobacco use, and treatment services. The association between smoking status and type of services received was tested using the Fisher’s exact test, considering a two-tailed test with p<0.05 as statistically significant. All analyses were conducted using R statistical software, version 4.1^7^.

## RESULTS

A total of 218 individuals completed a survey at the end of their clinic appointment. The average age of participants was 67 years, 49.5% (n=108) were female, 92.2% (n=201) were non-Hispanic White, and 62.4% (n=136) were high school graduates or higher. No significant differences were identified in demographic characteristics between the two groups.

### Determine the prevalence of tobacco use among patients being seen in a thoracic surgery and oncology clinic

Among the 218 participants who self-reported smoking status, 105 were in usual care (UC), and 113 in CEASE. As shown in Table 1, there were 27.6% (n=29) never smokers in UC and 27.7% (n=31) in CEASE; 60.0% (n=63) former smokers in UC compared to 50.9% (n=57) in CEASE; and 13 (12.4%) current smokers in UC compared to 21.4% (n=24) in CEASE. No differences in smoking status were noted between UC and CEASE (p=0.168).

**Table 1:** Pre- and post-implementation patient sociodemographics (N=218)

*Characteristics*	*Overall (N=218) n (%)*	*Usual care (pre) (N=105) n (%)*	*CEASE (post) (N=113) n (%)*	*p*
**Age** (years), median (IQR)	67.0 (18.0–99.0)	65.0 (35.0–87.0)	68.0 (18.0–99.0)	0.239
**Sex**				
Female	108 (49.5)	53 (50.5)	55 (48.7)	1.000
Male	104 (47.7)	52 (49.5)	52 (46.0)	
Missing	6 (2.8)	0 (0.0)	6 (5.3)	
**Ethnicity**				
Hispanic/Latino	7 (3.2)	1 (1.0)	6 (5.3)	0.056
Not Hispanic/Latino	194 (89.0)	104 (99.0)	90 (79.6)	
Missing	17 (7.8)	0 (0.0)	17 (15.0)	
**Race**				
Asian	3 (1.4)	3 (2.9)	0 (0.0)	0.104
Black/African American	3 (1.4)	0 (0.0)	3 (2.7)	
Other	3 (1.4)	1 (1.0)	2 (1.8)	
White/Caucasian	201 (92.2)	98 (93.3)	103 (91.2)	
Missing	8 (3.7)	3 (2.9)	5 (4.4)	
**Marital status**				
Married/partnered	130 (59.6)	61 (58.1)	69 (61.1)	0.398
Not married/partnered	82 (37.6)	44 (41.9)	38 (33.6)	
Missing	6 (2.8)	0 (0.0)	6 (5.3)	
**Work status**				
Not working/other	133 (61.0)	62 (59.0)	71 (62.8)	0.326
Working full/part-time	80 (36.7)	43 (41.0)	37 (32.7)	
Missing	5 (2.3)	0 (0.0)	5 (4.4)	
**Education level**				
Less than or high school	78 (35.8)	38 (36.2)	40 (35.4)	1.000
More than high school	136 (62.4)	67 (63.8)	69 (61.1)	
Missing	4 (1.8)	0 (0.0)	4 (3.5)	
**Income** ($)				
<50000	43 (19.7)	18 (17.1)	25 (22.1)	0.922
50000–99999	48 (22.0)	18 (17.1)	30 (26.5)	
≥100000	42 (19.3)	17 (16.2)	25 (22.1)	
Missing	85 (39.0)	52 (49.5)	33 (29.2)	
**Financial situation**				
Enough after bills	110 (50.5)	53 (50.5)	57 (50.4)	0.162
Enough to pay bills	46 (21.1)	29 (27.6)	17 (15.0)	
Pay bills after cutbacks	15 (6.9)	5 (4.8)	10 (8.8)	
Difficulty paying bills	6 (2.8)	3 (2.9)	3 (2.7)	
Missing	41 (18.8)	15 (14.3)	26 (23.0)	
Smoking status*				
Current/recent	37 (17.1)	13 (12.4)	24 (21.4)*	0.168
Former	120 (55.3)	63 (60.0)	57 (50.9)	
Never	60 (27.6)	29 (27.6)	31 (27.7)	

* One individual did not report smoking status. IQR: interquartile range.

### Determine the effectiveness of a computer-facilitated intervention in improving provider delivery of tobacco treatment in a thoracic surgery and oncology clinic

Of the 37 current smokers, there were significant differences in receipt of tobacco treatment by group. As shown in Table 2, 15.4% (n=2) of participants in UC and 62.5% (n=15) in CEASE received tobacco treatment services (Fisher’s exact test p<0.004). Two participants (15.4%) in UC reported receiving cessation resources, whereas those in CEASE reported receiving various types of tobacco treatment services including: 12.5% (n=3) quitline only, 8.3% (n=2) received medication only, 41.7% (n=10) received a combination of quitline plus medication, and 12.5% (n=3) did not answer the question (Fisher’s exact test p<0.002).

**Table 2:** Receipt of tobacco treatment among current smokers only (N=37)

*Treatment*	*Total (N=37) n (%)*	*Usual care (pre) (N=13) n (%)*	*CEASE (post) (N=24) n (%)*	p*
**No treatment**	17 (45.9)	11 (84.6)	6 (25.0)	0.004
**Missing**	3 (8.1)	0 (0.0)	3 (12.5)	
**Received treatment**	17 (45.9)	2 (15.4)	15 (62.5)	
Quitline only	3 (17.6)	0 (0.0)	3 (20.0)	0.002
Medication only	2 (11.8)	0 (0.0)	2 (13.3)	
Combination	10 (58.8)	0 (0.0)	10 (66.7)	
Other^a^	2 (11.7)	2 (100)	0 (0.0)	

a Two individuals selected ‘Other’ but did not specify the type of resources they received.

* Fisher’s exact test.

## DISCUSSION

The smoking prevalence rate in our study was 17% which is similar to the overall rate of smoking among adults in the United States but higher than those aged ≥65 years (8%)8. It is not surprising that smoking rates would be higher among patients seeking care in thoracic surgery and oncology care settings since lung cancer and other thoracic diseases have traditionally been associated with smoking. It is important to recognize, however, that 28% of participants were never smokers. The rate of never smokers seen within thoracic oncology settings has been increasing over the last decade from 13% to 28%^9^. This finding underscores that a systematic approach to screening and identification of current smokers is essential to initiating treatment in a busy practice setting.

Delivery of tobacco treatment is an essential part of routine care in thoracic surgery and oncology care settings, since patients who continue to smoke after thoracic surgery have increased pulmonary complications compared with never smokers (OR=3.31, p=0.007)^10^. Similarly, patients who continue smoking during cancer treatment versus those who quit smoking, experience worse survival (29 vs 20 months)^11^. Price et al.^12^ found that providers assess and advise patients to quit smoking but were substantially less likely to prescribe cessation medication. We found that a computer-facilitated intervention significantly increased provider delivery of tobacco treatment including prescription of cessation medication and referral for behavioral counseling. Our findings are similar to those of Satterfield et al.^13^ who found that the use of a computer-facilitated intervention increased delivery of tobacco treatment in a primary care setting. Moreover, Warren et al.^14^ found that only 39% of thoracic oncology providers actively provide cessation advice. In a more recent study examining current tobacco treatment practices in Accredited Cancer Programs of the American College of Surgeons, only 17.6 % prescribed medications^15^. In our study, more than half of patients received cessation medications as part of their tobacco treatment. The findings from our study provide an opportunity to increase implementation of tobacco treatment in thoracic surgery and oncology settings.

We used multiple implementation strategies to adapt and integrate the CEASE intervention into the practice setting. The strategies that we used are consistent with recent recommendations for implementing tobacco treatment in oncology settings which included: training stakeholders who can facilitate implementation in the clinical setting, changing the infrastructure to integrate the intervention into the workflow, supporting clinicians through use of educational resources, and developing key stakeholder relationships^16^. The benefit of leadership support, especially identifying a clinical champion to facilitate integration of the intervention into the workflow and providing support for prescribing cessation medication, is essential for enabling implementation of tobacco treatment^17^. The main findings of this study are summarized in Supplementary file Figure 1.

### Limitations

This study aimed to identify current smokers and initiate tobacco treatment within a busy clinical setting. A quasi-experimental design using a pre-post-test was used in this study. This type of design, measuring tobacco treatment before and after the intervention, was implemented and has several limitations that can affect the validity and generalizability of the results. Given that we did not have a proper randomization, it is difficult to attribute the observed changes solely to the intervention, as other external factors could also be responsible. Moreover, the lack of randomization can lead to selection bias, where the group receiving the intervention may differ systematically from the pre-intervention population. We tried to offset this limitation, however, by evaluating sequential participants for eligibility and enrollment into the study. Another limitation of the study was that we focused on improving the process of the delivery of tobacco treatment but did not assess smoking cessation outcomes. An important next step would be to assess whether initiation of tobacco treatment improved cessation rates.

## CONCLUSIONS

A computer-facilitated intervention improved the process of delivering evidence-based treatment in a thoracic surgery and oncology setting. We were able to more than double delivery of interventions to assist participants with cessation. This intervention provides a low-resource approach that has the potential to be scaled and implemented more broadly.

## Supplementary Material



## Data Availability

The data supporting this research are available from the authors on reasonable request.
