# Supplementary material for: Effectiveness of a computer-facilitated intervention on improving provider delivery of tobacco treatment in a thoracic surgery and oncology outpatient setting: A pilot study
Source: Tob Induc Dis. 2024 Apr 22;22:10.18332/tid/186272. doi: 10.18332/tid/186272 (PMC11033978; doi:10.18332/tid/186272)
Supplement: Supplementary file 1 [file TID-22-66-s1.pdf]

**Supplementary Material**

**Figure 1.** Main findings: A pre-post-test design to assess effectiveness of a computer facilitated intervention on improving provider delivery of tobacco treatment in a thoracic surgery and oncology outpatient setting, 2019 (N=218)

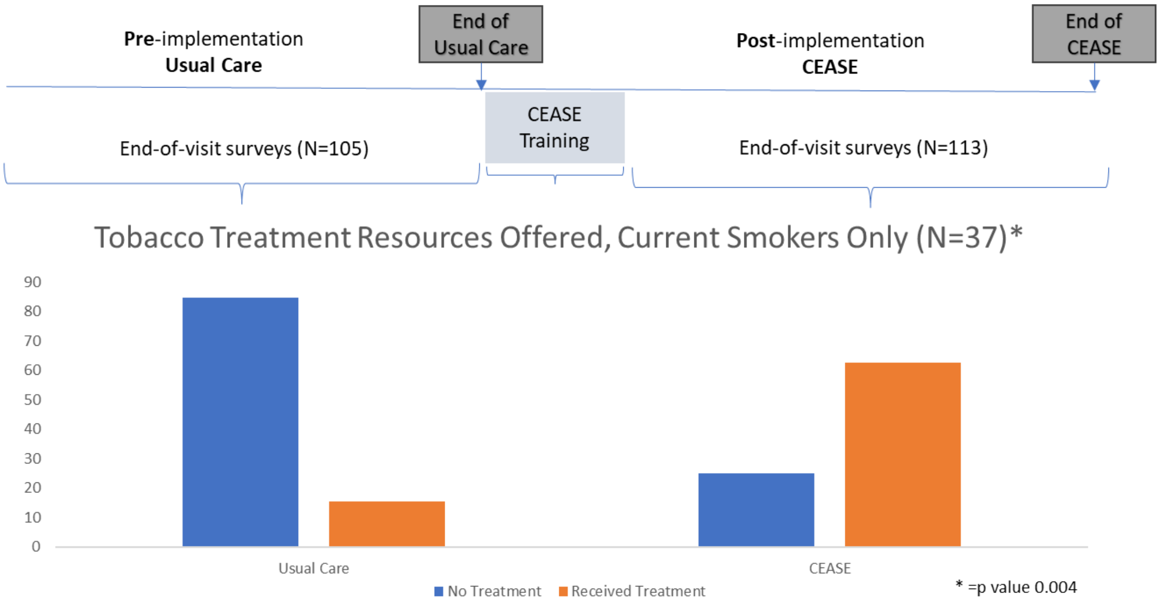


© **2024 Nayak M.M. et al.**
